# Comparison of short-pulse subthreshold (532 nm) and infrared micropulse (810 nm) macular laser for diabetic macular edema

**DOI:** 10.1038/s41598-020-79699-9

**Published:** 2021-01-08

**Authors:** Abdulrahman Al-Barki, Lamia Al-Hijji, Robin High, Patrik Schatz, Diana Do, Quan D. Nguyen, Jeffrey K. Luttrull, Igor Kozak

**Affiliations:** 1 King Khaled Eye Specialist Hospital, Riyadh, Saudi Arabia; 2 Department of Biostatistics, College of Public Health, University of Nebraska Medical Center, Omaha, NE USA; 3 Department of Clinical Sciences, Ophthalmology, Lund University, Skane University Hospital, Lund, Sweden; 4 Department of Ophthalmology, Byers Eye Institute, Stanford University, Palo Alto, CA USA; 5 Retina Diagnostic Laboratory of Ventura County, Ventura, CA USA; 6 Moorfields Eye Hospital Centre, Abu Dhabi, UAE

**Keywords:** Therapeutics, Oedema

## Abstract

The purpose of the study was to assess both anatomic and functional outcomes between short-pulse continuous wavelength and infrared micropulse lasers in the treatment of DME. This was a prospective interventional study from tertiary care eye hospital—King Khaled Eye Specialist Hospital (Riyadh, Saudi Arabia). Patients with center-involving diabetic macular edema were treated with subthreshold laser therapy. Patients in the micropulse group were treated with the 810-nm diode micropulse scanning laser TxCell (IRIDEX Corporation, Mountain View, CA, USA) (subthreshold micropulse—STMP group). Laser was applied according to recommendations for MicroPulse (125 microns spot size, 300 ms pulse duration and power adjustment following barely visible testing burn) in a confluent mode (low intensity/high density) to the entire area of the macular edema. Patients in the short-pulse group were treated with grid pattern laser with 20 ms pulse PASCAL laser 532 nm (TopCon Medical Laser Systems, Tokyo, Japan) with EndPoint algorithm, which was either 30% or 50% of testing burn (EndPoint 30% and EndPoint 50% groups, respectively). Main outcome measures included best-corrected visual acuity (BCVA in logMAR) and foveal thickness at baseline and the last follow-up visit at 6 months. There were 44 eyes in the micropulse group, 54 eyes in the EndPoint 50% group and 18 eyes in the EndPoint 30% group. BCVA for the whole cohort (logMAR) was 0.451 (Snellen equivalent 20/56) at baseline, 0.495 (Snellen equivalent 20/62) (p = 0.053) at 3 months, and 0.494 (Snellen equivalent 20/62) at the last follow-up (p = 0.052). Foveal thickness for the whole cohort was 378.2 ± 51.7 microns at baseline, 347.2 ± 61.3 microns (p = 0.002) at 3 months, and 346.0 ± 24.6 microns at the final follow-up (p = 0.027). As such the short-pulse system yields more temporary reduction in edema. Comparison of BCVA between baseline and 6 months for EndPoint 30%, EndPoint 50% and STMP groups was p = 0.88, p = 0.76 and p = 0.003, respectively. Comparison of foveal thickness between baseline and 6 months for EndPoint 30%, EndPoint 50% and STMP groups was p = 0.38, p = 0.22 and p = 0.14, respectively. We conclude that the infrared micropulse system seems to improve functional outcomes. When applied according to previously published reports, short-pulse system may yield more temporary reduction in edema while infrared micropulse system may yield slightly better functional outcomes.

## Introduction

Diabetic macular edema (DME) is the leading cause of moderate vision loss in patients with diabetes^1^. The role of laser photocoagulation, previously the mainstay of treatment of DME^2^, has undergone significant changes after the introduction of anti-vascular endothelial growth factor (anti-VEGF) therapy^3–5^. Three main areas of current laser use in DME include management of extrafoveal leakage in non-center involving macular edema, an adjunct management to intravitreal pharmacotherapy to reduce the frequency of required intravitreal injections for vision improvement and/or vision stabilization, and the use of non-damaging/tissue sparing photocoagulation techniques^3,6–10^.

Non-damaging retinal photocoagulation may be an effective treatment alternative to the modified Early Treatment Diabetic Retinopathy Study (ETDRS) macular photocoagulation technique, but without causing chorioretinal scarring or inducing visual field scotoma^10–12^. Two approaches are commonly mentioned both in clinical practice and in the scientific literature: short-pulse continuous wave (SPCW) and micropulsed laser treatments, both of which limit heat spread to adjacent retinal layers. “Subthreshold” delivery indicates an invisible laser application, and “micropulse” refers to a laser pulse in the microsecond range^10,13^. Published studies have shown clinical efficacy of subthreshold short-pulse^14,15^ as well as subthreshold micropulse (STMP)^16–21^ laser for DME. In the case of STMP, treatment can be done which is not only subthreshold clinically, but “truly” subthreshold, sublethal to the RPE^9,13^. However, there is no head-to-head comparison of patterned SPCW and STMP according to manufacturer recommended treatment guidelines. To fill this gap and enhance our knowledge and understanding of retina laser properties, we have conducted the following prospective study to compare the efficacy of SPCW and STMP in center-involved DME. The aim of the study was to assess both anatomic and functional outcomes in these two photocoagulation modalities.

## Results

### Patients

Overall, the patients included in this study are the ones who refused intravitreal injection therapy. Out of 93 consented patients who started prospective study, 65 had complete data at the end of study period. Fourteen patients had treatment in 1 eye and 51 patients had bilateral (116 eyes in total). Overall, there were 42 males and 23 females and 76 phakic, 38 pseudophakic and 2 aphakic eyes in the study (statistically significant difference between aphakic group and other groups). All patients had type 2 diabetes mellitus. Glycemia was poorly controlled (HbAC1 > 7.0%) in all patients (baseline: SPCW EndPoint 30% = 7.2%, SPCW EndPoint 50% = 7.3%, STMP = 7.2%; comparisons: EndPoint 30% vs EndPoint 50%: p = 0.22; EndPoint30% vs STMP: p = 0.22; EndPoint 50% vs STMP: p = 0.22). There were 13 treatment naïve patients and 52 patients had been previously treated with bevacizumab monotherapy (no intravitreal steroids) with last injection received more than 6 months from study baseline. There were 27 patients (44 eyes) in the micropulse group, 26 patients (54 eyes) in the EndPoint 50% group and 12 patients (18 eyes) in the EndPoint 30% group with mean ages of 59.4 ± 7.3, 63.4 ± 8.4 and 63.0 ± 6.3 years, respectively (no statistically significant difference). All treatments were performed without any complications. Mean follow-up period for the whole group was 8.13 ± 3.93 months; for STMP, and for SPCW EndPoint 50% and EndPoint 30% groups it was 9.5 ± 4.12, 6.97 ± 3.26 and 8.0 ± 4.37.0 months, respectively.

### Central foveal thickness

**Figure 1:**
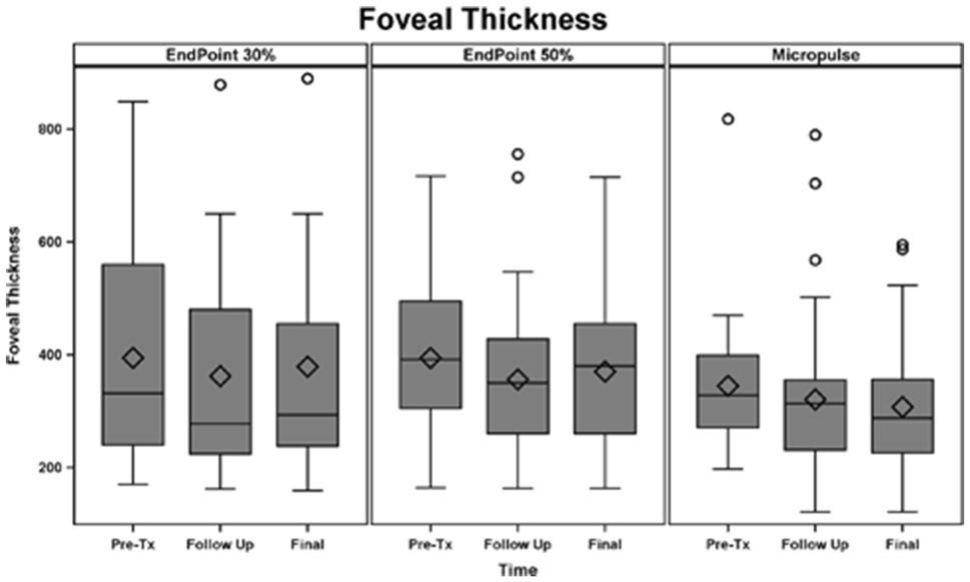
Graphic bar representation of central foveal thickness from baseline (pre-Tx), follow-up (3 months) to the last follow-up (6 months) among three laser treatment groups. Bars represent standard deviations, circles represent statistically significant difference among groups.

**Figure 2:**
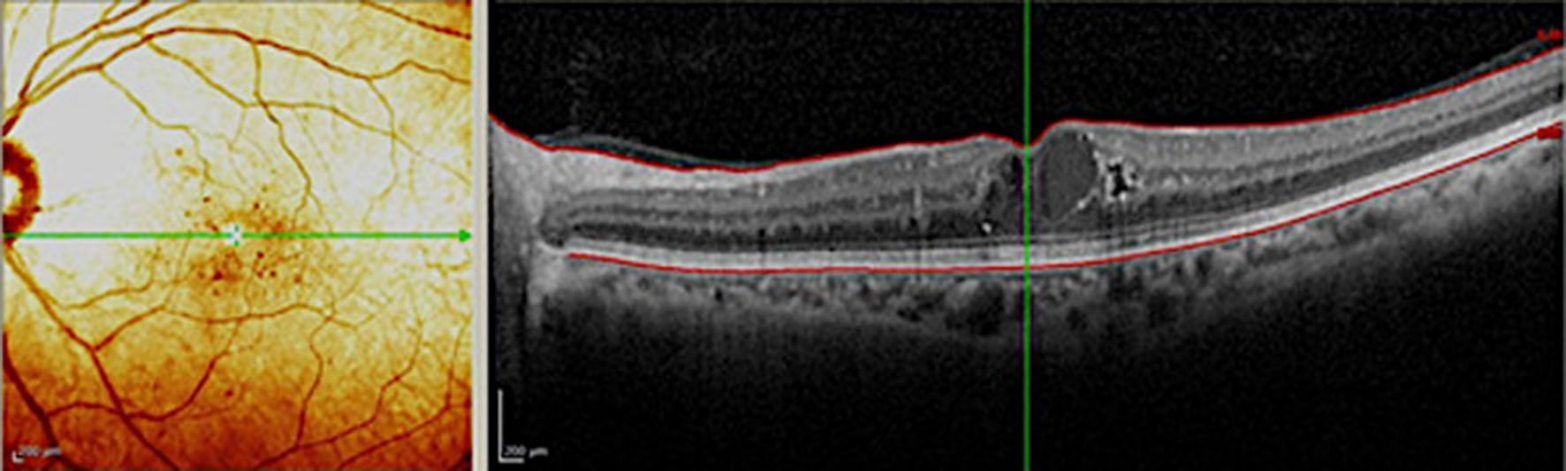
A pre-treatment spectral-domain optical coherence tomography scan of an eye with non-proliferative diabetic retinopathy and diabetic macular edema with increased foveal thickness.

**Figure 3:**
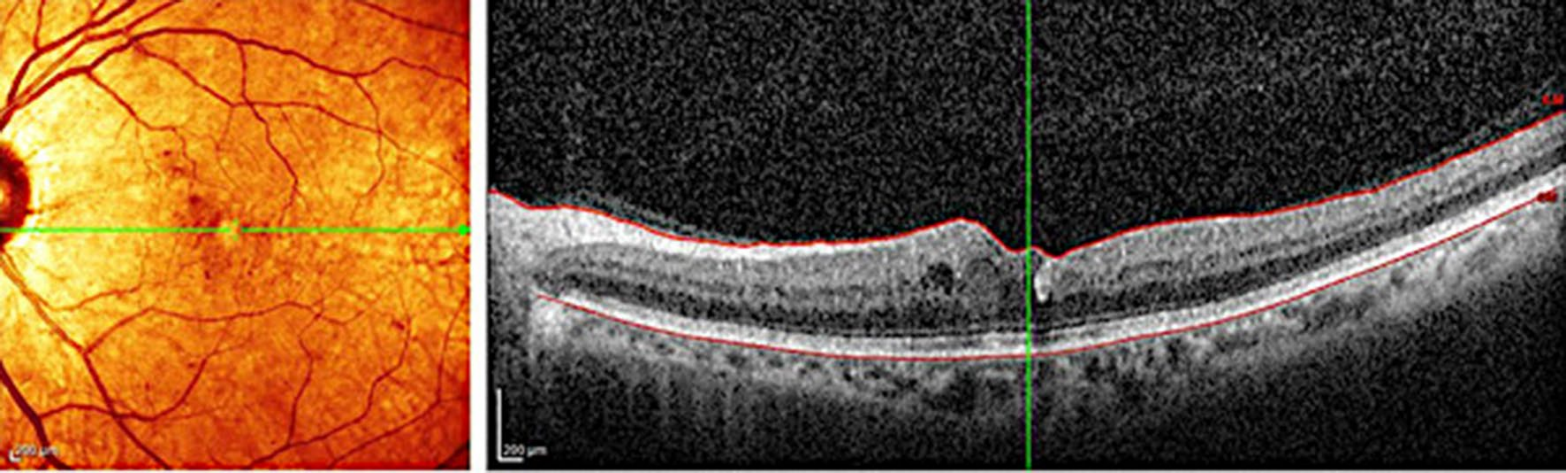
A post-treatment spectral-domain optical coherence tomography scan of the same eye following micropulse macular laser demonstrates improvement in diabetic macular edema and reduction of foveal thickness.

Foveal thickness for the entire cohort of all study subjects (116 eyes total) was 378.2 ± 51.7 microns at baseline; 347.2 ± 61.3 microns at 3 months (p = 0.002); and 346.0 ± 24.6 microns at the final follow-up (p = 0.027). Figure 1 depicts changes in foveal thickness after treatment in each treatment group (baseline: SPCW EndPoint 30% = 394.4 ± 201.5 microns, SPCW EndPoint 50% = 394.1 ± 128.5 microns and STMP = 344 ± 105.8 microns; comparisons—EndPoint 30% vs 50%: p = 0.61; EndPoint 30% vs STMP: p = 0.08; EndPoint 50% vs STMP: p = 0.09). Table 1 presents adjusted p-values for comparisons of foveal thickness at different time points in each treatment group. Foveal thickness was > 400 microns in 30 (25.8%) eyes before treatment. In the subanalysis of eyes with foveal thickness < 400 microns the results were similar than in the whole cohort (baseline: EndPoint 30% [273.3 ± 45.7 microns] vs EndPoint 50% [310.6 ± 55.8]: p = 0.06; EndPoint 30% vs STMP[298.9 ± 43.1]: p = 0.27; EndPoint 50% vs STMP: p = 0.11; at 3 months EndPoint 30% vs 50%: p = 0.22; EndPoint 30% vs STMP: p = 0.47; EndPoint 50% vs STMP: p = 0.77 and at 6 months EndPoint 30% vs 50%: p = 0.25; EndPoint 30% vs STMP: p = 0.38; EndPoint 50% vs STMP: p = 0.22). Figures 2 and 3 show representative images following STMP treatment.

**Table 1:** Adjusted p-values for comparisons of foveal thickness in eyes with diabetic macular edema at different time points among three treatment groups.

Laser	Pre-treatment versus 3 months	Pre-treatment versus 6 months	3 months versus 6 months
EndPoint 30%	0.18	0.38	0.99
EndPoint 50%	*0.005*	0.22	0.58
Micropulse	0.30	0.14	0.58

Statistically significant value is in italics

### Best-corrected visual acuity

**Figure 4:**
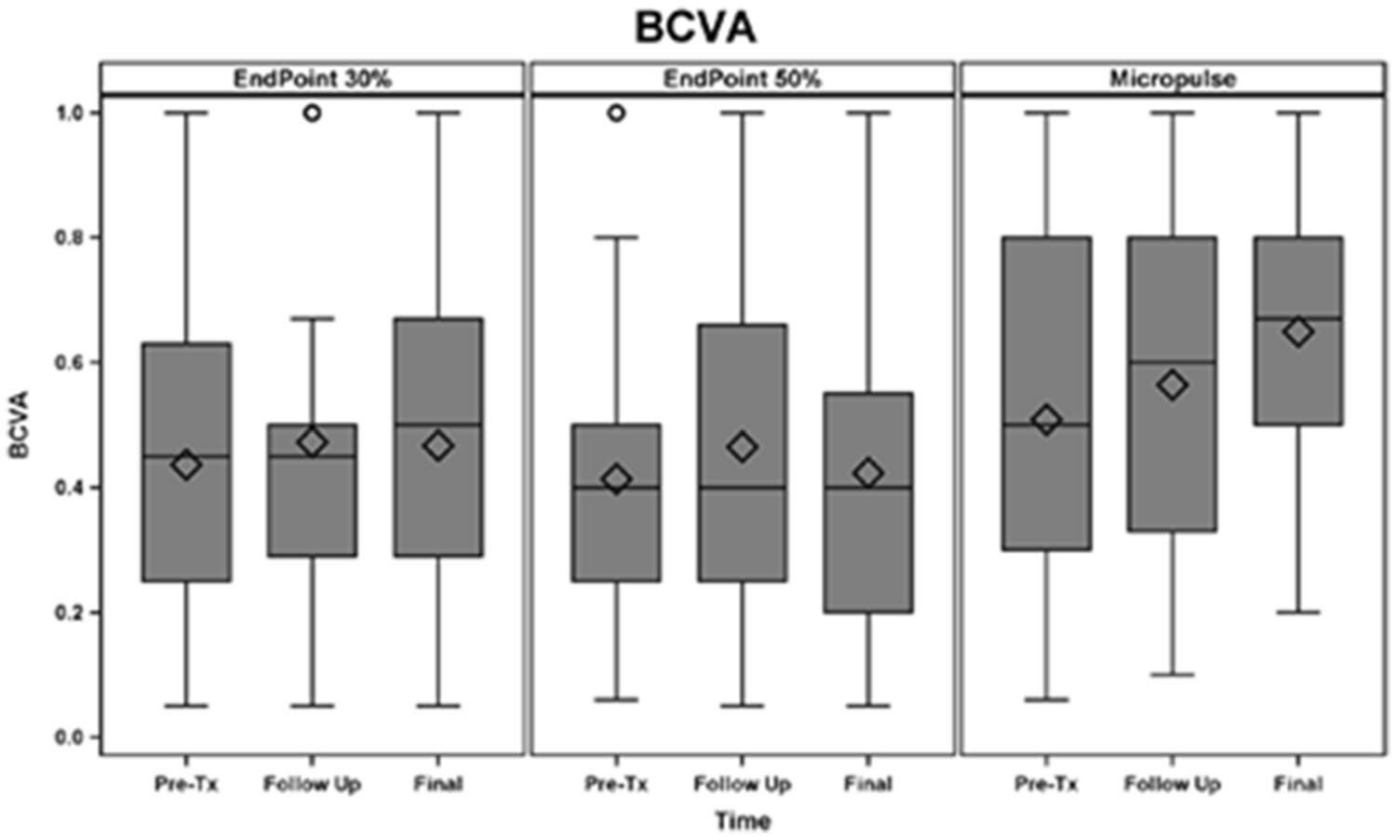
Graphic bar representation of best-corrected visual acuity (BCVA) from baseline (pre-Tx), follow-up (3 months) to the last follow-up (6 months) among three laser treatment groups. Bars represent standard deviations, circles represent statistically significant difference among groups.

BCVA for the whole cohort (logMAR) was 0.451 at baseline (Snellen equivalent 20/56), 0.495 (Snellen equivalent 20/62) at 3 months (p = 0.053) and 0.494 (Snellen equivalent 20/62) at the last follow-up (p = 0.052). Figure 4 depicts changes in BCVA after treatment in each treatment group (baseline: SPCW EndPoint 30% = 0.43 ± 0.2 (Snellen equivalent 20/52), SPCW EndPoint 50% = 0.41 ± 0.2 (Snellen equivalent 20/51) and STMP = 0.50 ± 0.2 (Snellen equivalent 20/61); comparisons—EndPoint 30% vs 50%: p = 0.3; EndPoint 30% vs STMP: p = 0.4; EndPoint 50% vs STMP: p = 0.4). Table 2 presents adjusted p-values for comparisons of best-corrected visual acuity at different time points in each treatment group.

**Table 2:** Adjusted p-values for comparisons of best-corrected visual acuity in eyes with diabetic macular edema at different time points among three treatment groups.

Laser	Pre-treatment versus 3 months	Pre-treatment versus 6 months	3 months versus 6 months
EndPoint 30%	0.53	0.88	0.75
EndPoint 50%	0.33	0.76	0.65
Micropulse	0.16	*0.003*	0.15

Statistically significant value is in italics

### Laser parameters, rescue injections and laser spot imaging

The mean number of laser shots was 148.3 ± 106.8, 165.7 ± 99.3 and 140.7 ± 66.1 in the STMP, and SPCW EndPoint 50% and EndPoint 30% groups, respectively. The mean laser power used was 340 ± 155 mW, 105 ± 48 mW and 125 ± 33 mW in the micropulse, EndPoint 50% and EndPoint 30% groups, respectively. At the end of final follow-up period, rescue therapy (intravitreal injections of antiangiogenic pharmacotherapeutic agents) were administered in 6 (13%), 14 (6%) and 4 (22%) eyes in the STMP, and SPCW EndPoint 50% and EndPoint 30% groups, respectively. None of the three pair-wise differences in the proportion of patients receiving rescue therapy were statistically significant. Table 3 presents adjusted p-values for comparisons of laser applications, power and need for rescue therapy at different time points in each treatment group. Table 4 presents data on presence of post-treatment laser scar in each group as imaged by color fundus photography and fundus autofluorescence.

**Table 3:** Adjusted p-values for comparisons of number of laser applications, power at different time points and need for rescue therapy (injections) in eyes with diabetic macular edema among three treatment groups.

Laser parameters	EndPoint 30% versus EndPoint 50%	EndPoint 30% versus micropulse	EndPoint 50% versus micropulse
Number of laser applications	0.94	0.99	0.96
Laser power	0.61	*0.007*	*0.001*
Need for rescue therapy	0.63	0.69	0.45

Statistically significant values are in italics

**Table 4:** Presence of post-treatment laser scar in eyes with diabetic macular edema in each treatment group as imaged by color fundus photography and fundus autofluorescence and graded anonymously.

Imaging	EndPoint 30% [eyes(%)]	EndPoint 50% [eyes(%)]	Micropulse [eyes(%)]
Color fundus photography	0 (0%)	3 (5.5%)	1 (2.2%)
Fundus autofluorescence	0 (0%)	3 (5.5%)	2 (4.5%)

## Discussion

Subthreshold macular laser treatment (thermal retinal photo-stimulation) is an appealing technique for treatment of diabetic macular edema if the clinician wishes to avoid traditional complications of conventional photocoagulation^12^. Its efficacy has been found comparable to conventional laser treatment of DME^10–12^. Our study reports on both anatomical and functional outcomes using suthreshold laser of two different techniques (SPCW and STMP) and wavelengths (532 nm versus 810 nm) among patients with similar ethnical background.

The mean reduction of foveal thickness for the entire cohort was 32 microns at the end of follow-up period, which reached statistical significance. Such reduction is comparable with other studies employing subthreshold laser photocoagulation for DME^15,18,22^. However, if we evaluate individual study arms, the only significant difference was observed between pre-treatment and month 3 follow-up time-point in the EndPoint 50% group. This observation is similar to what has been reported in other studies, reflecting the general consensus that if there is no improvement in macular edema by 6 months, it is unlikely that significant subsequent improvement will occur^11,16,17,23,24^.

It has been suggested that severity of macular edema influences the effects of subthreshold laser therapy, especially if foveal thickness is more than 400 microns^11^. Foveal thickness > 400 microns was present at baseline in one quarter of eyes in our cohort. The number of eyes with increased thickness was equally distributed across all treatment subgroups. Subgroup analyses of eyes with baseline foveal thickness > 400 microns, however, mirrored the results of the whole cohort.

While reduction in edema and decrease in foveal thickness are desired anatomic outcomes, previously conducted studies, including the ETDRS, have shown a weak correlation of central visual acuity to thickness^2,22,23^. In this study, visual acuity was significantly improved in only STMP eyes. The results are consistent with the findings of a meta analysis comparing STMP and conventional photocoagulation for DME^12^. High density of low intensity laser delivery, as used in this subgroup, has been reported to yield superior functional (visual) results compared to normal density treatment^12,16^ and could be why infrared subgroup showed more improvement. The high-density (application of confluent laser spots)/low-intensity (sublethal to the RPE) is intended to maximize treatment surface area, thus maximizing therapeutic recruitment of the retinal pigment epithelial cells and the therapeutic response to treatment^13^. The differences in number of laser applications in this study reflected the extend of macular edema. Significant visual acuity (VA) improvement did not occur during the first three months but between 4 to 6 months of follow-up, consistent with our knowledge that VA gain may lag behind anatomic improvement^12,13^.

There are two differences between our study design and previously published studies. While we followed the manufacturer’s recommendations regarding treatment parameters for STMP (125 microns spot size, 300 ms pulse duration and power adjustment following barely visible testing burn) and available reports at the beginning of our study^12^, the manufacturer’s treatment recommendations apply to approximately only 10% the treatments found to be most effective in published clinical studies^9,13,21,25^ Secondly, in our study, instead of re-treatment with laser after 3 months such as in other studies^16,26^ we observed the effect of laser treatment for 6 months before further intervention. This could be a reason for some drop-out of participants. While this protocol prolonged observation period for the initial effect of subthreshold laser, it was less aggressive compared to some current protocols, which may have aggravated any under-treatment, particularly in the STMP group. Lack of subjective improvement for this reason in some patients may have contributed to their loss to follow up. The decision to initiate rescue therapy with intravitreal injection of anti-angiogenic agents due to persistent DME was left to the discretion of the treating ophthalmologist. Several patients needed rescue therapy at the end of follow-up period but the proportion of those patients did not differ among three treatment groups. Whether laser retreatment would have been sufficient in these eyes is unknown.

We also observed the presence of mild laser scars, which were seen in 3 eyes on both fundus photos and fundus autofluorescence imaging in the EndPoint 50% subgroup. This may reflect the difficulty of finding the narrow 0.01 W wide therapeutic window of SPCW laser based on subjective assessment of the intensity of titration burns^13^. Additionally, scars were noted in 1 eye on fundus photo and in 2 eyes on fundus autofluorescence in the micropulse subgroup. Inadvertent retinal burns in micropulse laser therapy have been reported and are more likely to occur with 10% and 15% duty cycle^9,13^. We note that we followed the previously published reports and manufacturer’s recommended laser power titration recommendations for both SPCW and STMP. A review of the literature of subthreshold retinal laser treatment reveals that inadvertent retinal laser damage has been reported in every study employing power titration; and in no study using fixed laser parameters based on long clinical experience^9,21,26,27^. Laser scars following both yellow and diode micropulse macular treatment have been previously reported^17,28^. Histopathologic studies in animals by Yu et al., comparing micropulse 532 nm and high duty cycle (above 5%) 810 nm laser wavelengths showed damage to the RPE from both laser modes^29^. Thus, our study shares correlation in this regard with Yu, et al. and clinical comparisons of mETDRS photocoagulation with titrated high-duty cycle (15%), STMP for DME, which consistently demonstrate laser-induced retinal damage^15–17,19,20,28^.

The limitations of our study include non-randomized nature, which could introduce bias in the selection of treatment arm and relatively short follow-up period. We did not have the opportunity to measure other parameters of retinal function such as retinal sensitivity using microperimetry or multifocal electroretinography. Furthermore, we did not stratify patients according to glycemic control or diabetes duration, factors that are well-known to affect the risk of diabetes retinopathy, and thus may be associated with variable response to laser therapy. While the treatment adjustments were standardized, laser parameters always represent variable most difficult to control.

In summary, our study echoes previous reports finding comparable anatomic results from CW vs. MP laser, with better visual results following STMP laser^12^. Overall treatment effects in this cohort did not seem to be influenced by the degree of pre-treatment macular thickening. Non-responders treated by both laser systems may require similar proportion of rescue therapy. Both laser systems can produce inadvertent laser-induced retinal damage. Prior studies suggest this may be avoided in STMP by use of fixed laser parameters known to be both safe and effective in clinical practice, rather than the manufacturer’s recommended titration algorithm. Similarly, the literature also suggests that the results of STMP might have been improved by application of more laser spots, in higher density and over a larger area, compared to manufacturer recommendations. Due to the higher frequency of laser-induced retinal damage associated with the SPCW “endpoint management” titration algorithm, and inability to employ known safe and effective fixed CW laser parameters, attempts at improving the clinical results of SPCW by increasing treatment density would appear to be ill-advised, due to the risk of treatment-associated visual loss^10^. Larger, prospective, randomized studies of eyes with DME treated with subthreshold laser will help to elucidate further our understanding of subthreshold laser treatment.

## Materials and methods

The index study was a prospective, interventional, non-randomized study at the King Khaled Eye Specialist Hospital (KKESH) in Riyadh, Saudi Arabia from January 2015 to January 2016. The hospital KKESH Institutional Review Board granted permission to perform the study (Study #2015-PS-112), which adhered to the tenets of the Declaration of Helsinki and all research was performed in accordance with relevant guidelines and regulations. The trial has been registered (07/08/2020) as ClinicalTrials.gov Identifier: NCT04505306. Informed consent for treatment was obtained from all patients before study.

Inclusion criteria included center-involving clinically significant macular edema due to diabetic retinopathy (> 300 microns), clear ocular media, ETDRS visual acuity > 29 letters (Snellen equivalent of 20/150) or better, treatment naïve eyes or previously treated with antiangiogenic intravitreal agent(s) more than 6 months ago to allow for long wash-out period. All patients with prior antiangiogenic therapy had either recurrent edema or partial response to few injections in which they did not want to continue. As such, there were no cases of chronic, non-responsive DME defined as no change in foveal thickness or BCVA from previous visit lasting more than 6 months.

Exclusion criteria included non-center involving diabetic macular edema, previous retinal laser or surgery, intravitreal steroid use, and any condition that may be associated with a risk of macular edema such as age-related macular degeneration, retinal vein occlusion, vitreomacular traction, epiretinal membrane and others.

All patients had detailed ophthalmic examinations at baseline and follow-up visits at 3 and 6 months, including color fundus and fundus autofluorescence imaging (Topcon TRC-50DX, Topcon Medical Systems, Inc., NJ, US). The demographic data collection included age, gender, type of diabetes, eye laterality. The clinical data collection included best-corrected visual acuity (BCVA) measured using ETDRS vision charts, central foveal thickness, phakic status, laser power, duration, spot size, duty cycle, presence of laser scars on color fundus photographs and fundus autofluorescence and number of eyes requiring rescue treatment (injection) at the end of follow-up.

All patients had foveal thickness measurement using spectral-domain optical coherence tomography (SD-OCT) (Spectralis, Heidelberg Engineering, Heidelberg, Germany). The SD-OCT B-scan was based on the Spectralis macular raster consisting of 19 horizontals 6 mm line scans and a real-time eye tracking system. This enabled automated software algorithm to display with numeric averages of the macular thickness measurements for each of the 9 map sectors as defined by the Early Treatment Diabetic Retinopathy Study (ETDRS)^30^. Central retinal thickness was measured as the average thickness within 1 mm diameter centered around the fovea.

### Laser treatment

All treatments were performed by one physician (IK) using SPCW EndPoint 30% and 50% protocols and STMP laser. Area Centralis contact lens (× 1.06 magnification) after application of topical anesthesia was used in all patients. Patients in the STMP group were treated with the 810-nm diode micropulse scanning laser TxCell (IRIDEX Corporation, Mountain View, CA, USA) at 15% duty cycle. After detailed explanation of all techniques, the patients selected which treatment they wanted to receive. Laser was applied in the semi-confluent mode (low intensity/high density) to cover the entire area of the macular edema and leakage as imaged by OCT and/or fundus fluorescein angiography^13,18,21^. Patients in the SPCW group were treated with grid pattern laser with 20 ms pulse PASCAL laser 532 nm (TopCon Medical Laser Systems, Tokyo, Japan) with EndPoint algorithm, which was either 30% or 50% of testing burn with one burn width apart^16,31^. The OCT thickness and edema height on stereoscopic examination with contact lens determined selection of initial testing power. In both groups, subthreshold power was determined by titrating burn to light (barely visible) burn and switching to either micropulse mode with 15% duty cycle or 30% and 50% EndPoint value with automated power adjustment by laser machine. The power was not changed by the operator. EndPoint laser leaves barely visible spots at the corners of the treatment grid enabling approximated localization of laser application and avoidance of superimposition of the grids. Micropulse laser grid is entirely invisible. Laser spot size was 125 microns across the groups and fovea was spared to the area of 250 microns from the foveal center. Rescue treatment protocol allowed intravitreal antiangiogenic injection at month 6 if there was persistence of DME and no improvement in BCVA. Two independent graders who were masked to treatment arms evaluated images. In case of discrepancy, consensus was achieved with assistance of treating physician.

### Statistical analysis (repeated measures ANOVA with interaction)

Main outcome measures included best-corrected visual acuity (BCVA) and foveal thickness at baseline and the last follow-up visit. Secondary outcomes included need for rescue therapy and safety observations. Each study subject contributed up to three data points from one or two eyes. These data were first assessed graphically for outliers. With a continuous and unbounded (i.e., no fixed boundaries exist) response variable, the relevant statistical analysis is a two-way repeated measures ANOVA model which consists of one between subject factor (group) and two within subjects factors: eye (OD/OS) and time (baseline, month 3 and 6). These factors were evaluated as fixed effects. Their main effects and interactions were evaluated with type III tests of statistical significance. To work with the repeated measures, a special type of unstructured covariance matrix for the two within subject factors was applied^32^. When a significant interaction of eye and time was present, comparisons of factor means were evaluated for one of the factors at fixed levels of the other factor. In this analysis, differences over time were evaluated with a paired T-test having a variance/covariance matrix determined by the overall study design. To account for multiple comparisons between group means, adjustments to the p-values and confidence intervals for the differences were computed with simulation techniques^33^.

All statistical significance tests were two-sided. Statistical analyses were generated with PROC GLIMMIX from SAS/STAT software, Version 9.4 (2002–2012) of the SAS System for Windows (Cary, NC, USA).

## References

[1] Klein R, Moss SE, Klein BE, Davis MD, DeMets DL (2002). The Wisconsin epidemiologic study of diabetic retinopathy XI. The incidence of macular edema. Ophthalmology.

[2] Early Treatment Diabetic Retinopathy Study Research Group (1985). Photocoagulation for diabetic macular edema. Arch. Ophthalmol..

[3] Nguyen QD, Shah SM, Khwaja AA, Channa R, Hatef E, Do DV, Boyer DS, Heier JS, Abraham P, Thach AB, READ-2 Study Group (2010). Two-year outcomes of the ranibizumab for edema of the macula in diabetes (READ-2) study. Ophthalmology.

[4] Do DV, Nguyen QD, Boyer D, Schmidt-Erfurth U, Brown DM, Vitti R, Berliner AJ, Gao B, Zeitz O, Ruckert R, da Vinci Study Group (2012). One-year outcomes of the da Vinci study of VEGF trap-eye in eyes with diabetic macular edema. Ophthalmology.

[5] Wells JA, Glassman AR, Ayala AR, Jampol LM, Aiello LP, Antoszyk AN, Arnold-Bush B, Baker CW, Bressler NM, Browning DJ, Diabetic Retinopathy Clinical Research Network (2015). Aflibercept, bevacizumab, or ranibizumab for diabetic macular edema. N Engl J Med..

[6] Barteselli G, Kozak I, El-Emam S, Chhablani J, Cortes MA, Freeman WR (2014). 12-month results of the standardized combination therapy for diabetic macular oedema: Intravitreal bevacizumab and navigated retinal photocoagulation. Br. J. Ophthalmol..

[7] Framme C, Walter A, Prahs P, Theisen-Kunde D, Brinkmann R (2008). Comparison of threshold irradiances and online dosimetry for selective retina treatment (SRT) in patients treated with 200 nanoseconds and 1.7 microseconds laser pulses. Lasers Surg. Med..

[8] Framme C, Brinkmann R, Birngruber R, Roider J (2002). Autofluorescence imaging after selective RPE laser treatment in macular diseases and clinical outcome: A pilot study. Br. J. Ophthalmol..

[9] Luttrull JK, Sramek C, Palanker D, Spink CJ, Musch DC (2012). Long-term safety, high-resolution imaging, and tissue temperature modeling of subvisible diode micropulse photocoagulation for retinovascular macular edema. Retina.

[10] Chehade L, Chidlow G, Wood J, Casson RJ (2016). Short-pulse duration retinal lasers: A review. Clin. Exp. Ophthalmol..

[11] Mansouri A, Sampat KM, Malik JN, Glaser BM (2014). Efficacy of subthreshold micropulse laser in the treatment of diabetic macular edema is influenced by pre-treatment central foveal thickness. Eye.

[12] Chen G, Tzekov R, Li W, Jiang F, Mao S, Tong Y (2016). Subthreshold micropulse diode laser versus conventional laser photocoagulation for diabetic macular edema: A meta-analysis of randomized controlled trials. Retina.

[13] Luttrull JK, Dorin G (2012). Subthreshold diode micropulse laser photocoagulation (SDM) as invisible retinal phototherapy for diabetic macular edema: A review. Curr. Diabetes Rev..

[14] Jain A, Collen J, Kaines A, Hubschman JP, Schwartz S (2010). Short-duration focal pattern grid macular photocoagulation for diabetic macular edema: Four-month outcomes. Retina.

[15] Pei-Pei W, Shi-zhou H, Zhen T, Lin L, Ying L, Jiexiong O, Wen-Bo Z, Chen-Jin J (2015). Randomised clinical trial evaluating best-corrected visual acuity and central macular thickness after 532-nm subthreshold laser grid photocoagulation treatment in diabetic macular edema. Eye.

[16] Lavinsky D, Cardillo JA, Melo LA, Dare A, Farah ME, Belfort R (2011). Randomized clinical trial evaluating mETDRS versus normal or high-density micropulse photocoagulation for diabetic macular edema. Investig. Ophthalmol. Vis. Sci..

[17] Figueira J, Khan J, Nunes S, Sivaprasad S, Rosa A, de Abreu JF, Cinha-Vaz JG, Chong NV (2009). Prospective randomized controlled trial comparing sub-threshold micropulse diode laser photocoagulation and conventional green laser for clinically significant diabetic macular oedema. Br. J. Ophthalmol..

[18] Vujosevic S, Bottega E, Casciano M, Pilotto E, Convento E, Midena E (2010). Microperimetry and fundus autofluorescence in diabetic macular edema: Subthreshold micropulse diode laser versus modified early treatment diabetic retinopathy study laser photocoagulation. Retina.

[19] Kwon YH, Lee DK, Kwon OW (2014). The short-term efficacy of subthreshold micropulse yellow (577-nm) laser photocoagulation for diabetic macular edema. Korean J. Ophthalmol..

[20] Inagaki K, Ohkoshi K, Ohde S, Deshpande GA, Ebihara N, Murakami A (2015). Comparative efficacy of pure yello (577-nm) and 810-nm subthreshold micropulse laser photocoagulation combined with yellow (561–577-nm) direct photocoagulation for diabetic macular edema. Jpn. J. Ophthalmol..

[21] Vujosevic S, Martini F, Longhin E, Convento E, Cavarzeran F, Midena E (2015). Subthreshold micropulse yellow laser versus subthreshold micropulse infrared laser in center-involving diabetic macular edema: Morphologic and functional safety. Retina.

[22] Browning DJ, Glassman AR, Aiello LP, Bressler NM, Bressler SB, Danis RP, Davis MD, Ferris FL, Huang SS, Kaiser PK, Diabetic Retinopathy Clinical Research Network (2008). Optical coherence tomography measurements and analysis methods in optical coherence tomography studies of diabetic macular edema. Ophthalmology.

[23] Browning DJ, Glassman AR, Aiello LP, Diabetic Retinopathy Clinical Research Network (2007). Relationship between optical coherence tomography-measured central retinal thickness and visual acuity in diabetic macular edema. Ophthalmology.

[24] Laursen ML, Moeller F, Sander B, Sjoelie AK (2004). Subthreshold micropulse diode laser treatment in diabetic macular oedema. Br. J. Ophthalmol..

[25] Luttrull JK, Spink CJ (2006). Serial optical coherence tomography of subthreshold diode laser micropulse photocoagulation for diabetic macular edema. Ophthal. Surg. Lasers Imaging.

[26] Luttrull JK, Musch DC, Mainster MA (2005). Subthreshold diode micropulse photocoagulation for the treatment of clinically significant diabetic macular edema. Br. J. Ophthalmol..

[27] Luttrull JK, Sinclair SD (2014). Safety of transfoveal subthreshold diode micropulse laser for intra-foveal diabetic macular edema in eyes with good visual acuity. Retina.

[28] Chhablani J, Alshareef R, Kim DT (2018). Comparison of different settings for yellow subthreshold laser treatment in diabetic macular edema. BMC Ophthalmol..

[29] Yu AK, Merrill KD, Truong SN, Forward KM, Morse LS, Telander DG (2013). The comparative histologic effects of subthreshold 532- and 810-nm diode micropulse laser on the retina. Investig. Ophthalmol. Vis. Sci..

[30] Early Treatment Diabetic Retinopathy Study Research Group (1991). ETDRS report number 7: Early treatment diabetic retinopathy study design and baseline patient characteristics. Ophthalmology.

[31] Lavinsky D, Sramek C, Wang J (2014). Subvisible retinal laser therapy: Titration algorithm and tissue response. Retina.

[32] Littell RC, Milliken GA, Stroup WW, Wolfinger RD, Schabenberger O (2006). SAS for Mixed Models.

[33] Westfall P, Tobias RD, Wolfinger RD (2011). Multiple Comparisons and Multiple Tests Using SAS@R.

